# Gp130-Dependent Release of Acute Phase Proteins Is Linked to the Activation of Innate Immune Signaling Pathways

**DOI:** 10.1371/journal.pone.0019427

**Published:** 2011-05-04

**Authors:** Maren Luchtefeld, Christoph Preuss, Frank Rühle, Eskindir P. Bogalle, Anika Sietmann, Stefanie Figura, Werner Müller, Karsten Grote, Bernhard Schieffer, Monika Stoll

**Affiliations:** 1 Department of Cardiology and Angiology, Hannover Medical School, Hannover, Germany; 2 Genetic Epidemiology of Vascular Disorders, Leibniz-Institute for Arteriosclerosis Research, Muenster, Germany; 3 Faculty of Life Sciences, University of Manchester, Manchester, United Kingdom; Oregon Health and Science University, United States of America

## Abstract

**Background:**

Elevated levels of acute phase proteins (APP) are often found in patients with cardiovascular diseases. In a previous study, we demonstrated the importance of the IL-6-gp130 axis -as a key regulator of inflammatory acute phase signaling in hepatocytes-for the development of atherosclerosis.

**Background/Principal Findings:**

Gp130-dependent gene expression was analyzed in a previously established hepatocyte-specific gp130 knockout mouse model. We performed whole transcriptome analysis in isolated hepatocytes to measure tissue specific responses after proinflammatory stimulus with IL-6 across different time points. Our analyses revealed an unexpected small gene cluster that requires IL-6 stimulus for early activation. Several of the genes in this cluster are involved in different cell defense mechanisms. Thus, stressors that trigger both general stress and inflammatory responses lead to activation of a stereotypic innate cellular defense response. Furthermore, we identified a potential biomarker Lipocalin (LCN) 2 for the gp130 dependent early inflammatory response.

**Conclusions/Significance:**

Our findings suggest a complex network of tightly linked genes involved in the early activation of different parts of the innate immune response including acute phase proteins, complement and coagulation cascade.

## Introduction

Atherosclerosis is a major cause of cardiovascular disease worldwide and characterized by artery wall thickening due to a chronic inflammation in the intima of the arteries leading to atherosclerotic plaque formation in the vessel wall [1],[2].

Unfortunately, strategies targeting key inflammatory mediators had only limited success so far, highlighting the complex pathogenesis of the disease. The inflammatory process in the vessel wall is associated with enhanced levels of acute phase proteins (APP), which are mainly expressed in the liver [3]. These proteins are regarded as important components of the innate immune response and powerful predictors of cardiovascular incidences [4].

Although the acute phase response has been in focus of investigation for many years [5], several aspects regarding the functional implication of APP release in the process of chronic inflammation are still not fully understood.

In an earlier study, we demonstrated the importance of the gp130 cytokine receptor as a key regulator of acute phase signaling is crucially involved in atherosclerotic plaque formation in a mouse model of experimental atherosclerosis. Besides this important role of the gp130 signal transducer, the cytokine IL-6 is widely considered as the major inducer of APP production in mice hepatocytes. Interestingly, IL-6 deficient mice exhibit only a partly impaired APP production which suggests a redundant function for the IL-6 cytokine family of cytokines which share the common signalling receptor gp130 [5], [6].

In order to better define the role of the IL-6 cytokines in the gp130 knockout mouse model, we analyzed gp130 dependent gene expression in isolated hepatocytes using a whole transcriptome approach. Although local effects of gp130 dependent down-regulation have been described [7], we aimed to study the consequences of gp130-deficiency in our model system in a broader context using a microarray time-course experiment. Gp130-deficient hepatocytes cells were stimulated with IL-6 to induce an inflammatory response. Changes in expression status compared to IL-6-stimulated control cells from wild type mice were measured for five consecutive time points (0 h, 1 h, 3 h, 6 h, and 12 h). In contrast to resected liver tissue samples, this approach provides a more accurate in-vitro model of studying the liver specific response after a proinflammatory stimulus.

While proinflammatory factors are considered key elements in the pathogenesis of chronic inflammatory diseases, the underlying mechanisms remain enigmatic [8]. Therefore, the goal of this study was not only to target key inflammatory factors but also to highlight correlated transcriptional responses to IL-6 treatment which is in turn linked to chronic inflammation. A tight control of the complex regulatory pathways is critical for immune homeostasis and tissue specific gene expression. Growing evidence highlighted the role of gp130-dependent gene expression in the liver as critical for the regulation of the innate immune response. A recent study demonstrated that the gp130 system controls innate immune responses during infection by promoting myeloid-derived suppressor cell function [9].

In this study, we report that a surprisingly small cluster of 8 gp130-dependent genes show a correlated transcriptional response after IL-6 stimulation in hepatocytes. We identified differentially expressed genes that play an important role as markers and mediators of chronic inflammation. Co-expression network analysis and pathway mapping revealed that these genes share a functional role. The stimulatory IL-6 action overlaps with hepatic acute-phase and innate immune responses. This might link the gp130 system with the recruitment and activation of innate immune cells as central players for inflammatory disorders including cardiovascular diseases such as atherosclerosis.

## Results

### Correlated response of gp130-dependent genes expression

In order to better define the role of the hepatic acute phase response during inflammation and development of atherosclerosis we used a hepatocyte-specific gp130 knockout model. Based on our previous findings, we aimed to identify novel liver derived gp130 dependent factors in mice, which might play an essential role in chronic inflammatory processes. Therefore, we performed transcriptome-wide analysis of 26,766 genes by microarray time-course analysis of isolated hepatocytes. The transcriptome profiling revealed a surprisingly small number of 38 genes that are differentially expressed with a p-value of <0.05 and >1.5 fold after IL-6 stimulation in at least one of the five time points (Table S1).

**Figure 1:**
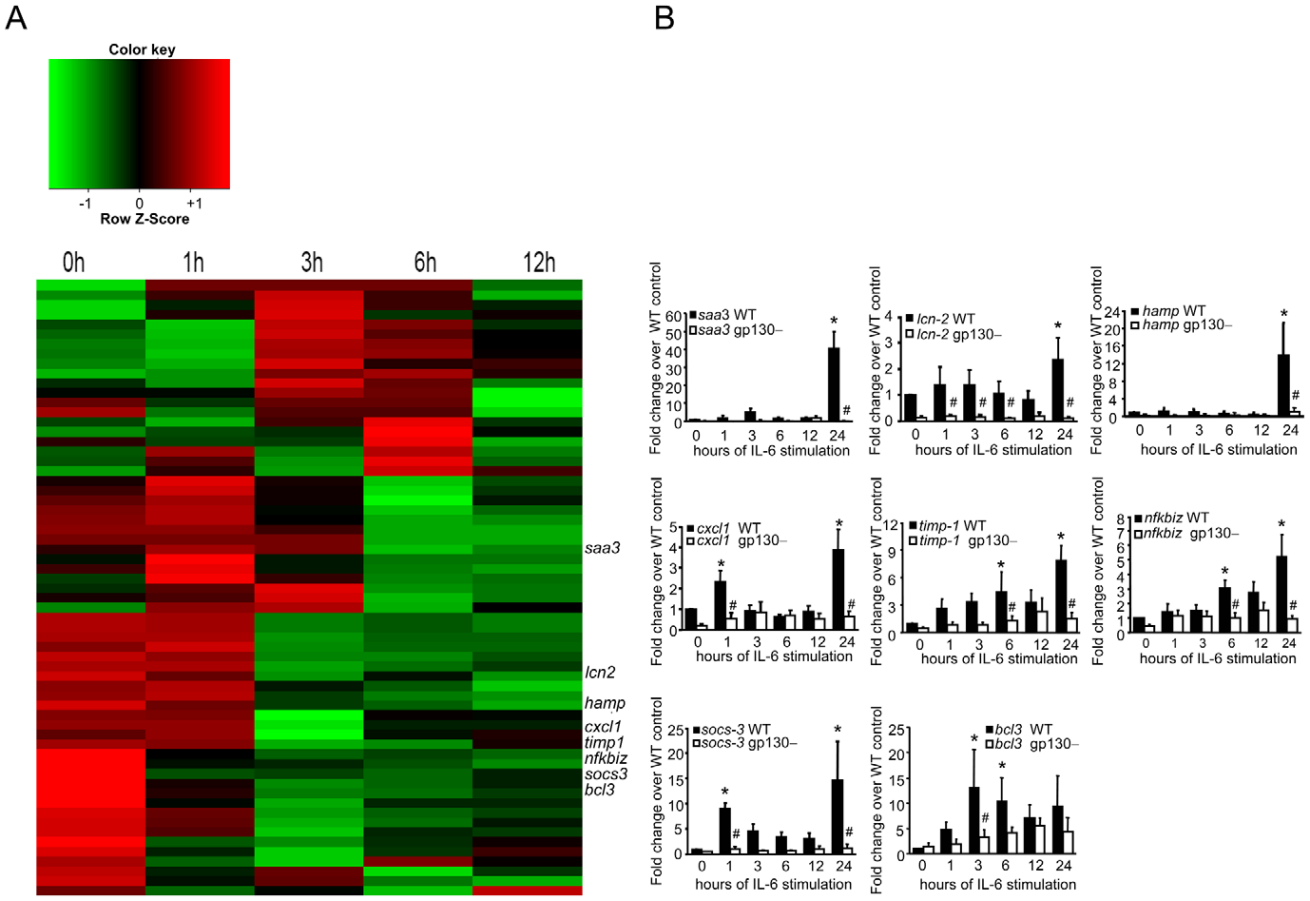
Co-expression analysis of gp130-regulated genes. (A) Time-course gene microarray analysis of hepatocytes for five different time points before and after IL-6 stimulation (n>5 per group). Genes with >1.5 and <−1.5 fold change after IL-6 stimulation were clustered in a heatmap diagram. Red indicates up-regulation compared with wild-type mice and green indicates down-regulation. (B) Quantitative real-time PCR of hepatocytes taken from untreated (control), gp130–and wildtype (WT; gp130 flox) mice after time-dependent IL-6 stimulation. * P<0.05. Data are presented as mean±SEM of at least 8 independent experiments.

Instead of focusing on the analysis of single differentially expressed genes, we were interested in identifying a cluster of genes sharing a coordinated response after IL-6 stimulation in the hepatocyte-specific gp130 mouse model. Hierarchical clustering of the time course expression data revealed that a group of 8 gp130 dependent genes showed a coordinated early response to stimulation with IL-6 in hepatocytes. A heatmap diagram displays the group of genes that are similarly regulated in Figure 1A. The group includes: (1) acute phase proteins including *saa3* and *lcn2* (2) chemokines with cytokine-cytokine interactions, such as *cxcl1* and (3) extracellular matrix proteins, such as *timp-1*. The coordinated expression patterns of these gp130 dependent genes point to a common underlying mechanism responsible for the early inflammatory response in IL-6 stimulated hepatocytes. Of the genes that are subject to synergistic activation by IL-6, the *lcn2* gene showed the most striking potentiation. The results of the microarray data for this group of genes were then further confirmed by real-time PCR (Figure 1 B).

### Validation of soluble proteins for the gp130 model of atherosclerosis

Gene co-expression analysis provides powerful information to identify new functionally related genes in a tissue specific context. However, this expression relationship only reflects mRNA transcripts but not protein levels. Therefore, we tested the soluble gene products of the correlated cluster harboring 8 genes identified through transcriptome profiling in hepatocyte supernatant to measure differences in soluble protein levels.

**Figure 2:**
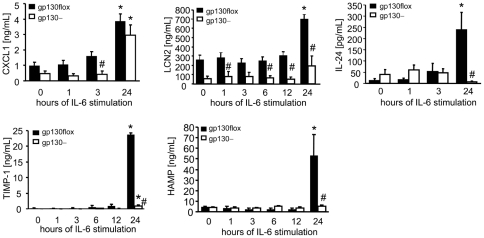
Analysis of gp-130 regulated soluble factors. CXLC1, LCN2, TIMP-1, HAMP and IL-24 concentration in the supernatant of IL-6 stimulated hepatocytes. Stimulation with IL-6 for 24 hours induces the release of CXCL1, LCN2, TIMP-1, HAMP and IL-24 within the supernatant of WT (gp130 flox)-derived hepatocytes (CXCL1, LCN2, TIMP-1, HAMP and IL-24) or gp130–-derived hepatocytes (CXCL1, TIMP-1). CXCL1 was found to be differentially regulated after 3 hours of IL-6 stimulation, LCN2 1, 6, 12 and 24 hours after IL-6 stimulation TIMP-1 and HAMP after 24 hours after IL-6 stimulation. Data are presented as mean±SEM of at least 4–5 independent experiments.

Since LCN 2, CXLC2, TIMP-1, HAMP and IL-24 protein expression is also induced to be released in the supernatant of hepatocytes upon IL-6 stimulation in a gp130-dependent manner, as confirmed by ELISA technique (Figure 2). Next we investigated whether these 8 genes in hepatocytes and the related soluble factors may be the onset of experimental atherosclerosis in a mouse model. Therefore, we compared gp130–/apoE– mice compared to apoE–mice fed a high-fat western type diet for 12 weeks. Accordingly mRNA expression was investigated within the liver, and protein levels were analyzed in the plasma of these mice.

**Figure 3:**
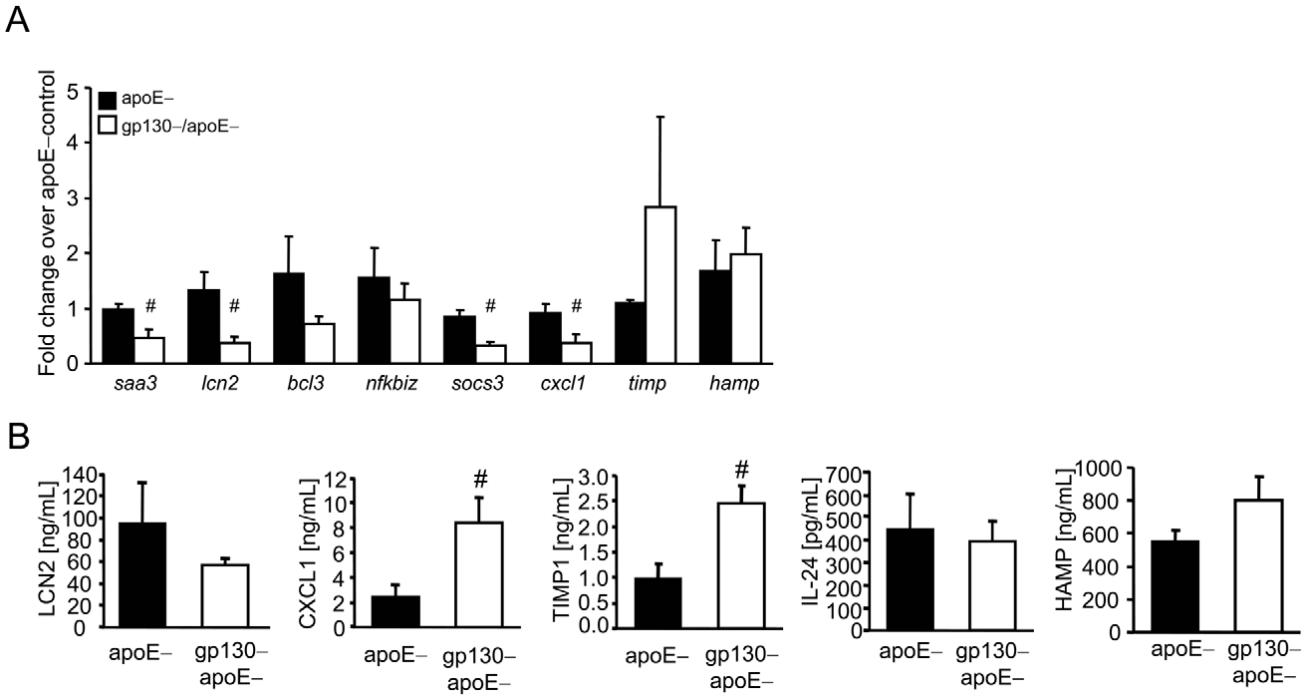
Analysis of gp130-regulated genes and soluble factors in a mouse model of atherosclerosis. (A) Quantitative real-time PCR of livers (n = 4–6 per genotype) taken from gp130–/apoE– and apoE– mice following feeding a cholesterol rich diet for 12 weeks. *Saa3*, *lcn2*, *socs3* and *cxcl1* mRNA-expression were significantly reduced in gp130-/apoE- mice compared to apoE-mice. (B) CXCL1, LCN2, TIMP-1, HAMP and IL-24 plasma levels from gp130–/apoE– and apoE– mice following feeding a cholesterol rich diet for 12 weeks. CXCL1 and TIMP-1 plasma levels are significantly increased in *gp130*–/*apoE*–- mice compared to *apoE*–mice, whereas LCN2 plasma levels are reduced within the same mice. IL-24 and HAMP plasma levels did not differ between the genotypes. Data are presented as mean±SEM of at least 6–10 mice/genotype; *P<0.05 vs. control, #p<0.05 vs. genotype.

Significant differences in mRNA expression were observed for *saa3, lcn2, socs-3 and cxcl-1 genes*, and differences in protein levels were observed for *cxcl1*, *timp-1* and *lcn2* genes (Figure 3). CXCL1 mRNA expression was reduced in the liver of gp130–/apoE–mice compared to apoE–mice, whereas plasma levels were significantly increased. Thus it is tempting to speculate that other cells then hepatocytes e.g. macrophages, neutrophils or epithelial cells [13] are responsible for an increase of CXCL1 in the plasma. Of interest, we observed a significant increase of TIMP-1 mRNA-expression in the liver and in the plasma of gp130–/apoE– mice, compared to apoE– mice. An IL-6 dependent TIMP-1 induction was recently demonstrated in a hepatoma cell line [14]. Since we investigated TIMP-1 expression in the whole liver, we cannot exclude–that besides hepatocytes–other hepatic cells such as endothelial cells and Kupffer cells which are minor cell populations in the liver, also contribute to this effect.

Only LCN-2 was found to be reduced in the plasma of gp130–/apoE– mice, which also showed the statistical strongest signs of differential gene expression across all measured time points. For the remaining candidate genes, only slight changes in protein levels were observed in hepatocyte supernatants.

### Pathway mapping

KEGG pathway mapping was used to search for over-represented classes of gene functions for the identified 38 genes. This was done by comparison of d.e genes for each time point with canonical KEGG pathways using the hypergeometric distribution function implemented in the statistical language R.

Over-represented pathways were detected for all time-points after IL-6 stimulation. We could identify gp130-regulated genes, which are associated with the activation of the JAK/STAT (*socs-3*, *Il-13ra1*, *cish*, *osmr*, *Il-24*), and the cytokine signalling pathway (*Il-13ra1*; *osmr*, *cxcl1*). Among both pathways, which play an important role in the inflammatory response, 4 members of the co-expressed cluster were found (*socs3*, *cxcl1*, *Il-13ra1*, *cish*). Table 1 displays the pathways, according to the number of time points and membership of d.e genes.

**Table 1:** KEGG pathways overrepresented by IL-6-gp130 gene expression.

Timepoint	KEGG ID	Pathway	Input genes	p-value
0 h	-	-	-	-
1 h	mmu04630	Jak-STAT signaling pathway	*Il-24*, *socs3*	5.4×10^−4^
3 h	mmu04060	Cytokine-cytokine receptor interaction	*Il-24*, *Il13 ra1*	6.5×10^−3^
	mmu04630	Jak-STAT signaling pathway	*Il-24*, *socs3*, *Il13ra1,cish*	1×10^−5^
6 h	mmu04060	Cytokine-cytokine receptor interaction	*osmr*, *Il-24*, *Il-13 ra1*	9.7×10^−4^
	mmu04630	Jak-STAT signaling pathway	*osmr* ,*Il-24*, *socs3*, *Il13 ra1*, *cish*	1×10^−5^
12 h	mmu04060	Cytokine-cytokine receptor interaction	*osmr*, *Il-24*, *Il-13ra1*, *cxcl1*	7×10^−5^
	mmu04630	Jak-STAT signaling pathway	*osmr*, *Il-24*, *socs3*, *Il13 ra1*	1×10^−5^

KEGG-database analysis revealed several gp130-regulated genes which are time-dependently associated with JAK/STAT (*Il-24*, *socs3*, *Il-13ra1*, *cish*, *osmr*) and cytokine signalling pathway (*Il-13ra1*, *Il-24*, *osmr*, *cxcl1*).

### Co-expression network analysis

Conventional annotation methods measuring the over-representation of functional categories, such as the performed KEGG-pathway mapping may lack an adequate representation of expression data in the functional specific cellular context. For this reason, we performed a co-expression network analysis to reveal in which functional cellular context the cluster with the 8 genes was expressed.

Co-expression network analysis is based on the idea that large collections of microarray data contain information about concerted changes in transcript levels. Genes which share a functional role are often found to be co-expressed in the same molecular network which in turn is driven by the same underlying regulatory factors.

In order to test the hypothesis that liver-derived gp130 dependent factors are involved in the same underlying molecular network and to identify new functionally related genes, we used co-expression data derived from a set of publicly available microarray experiments in mice from the Coxpresdb database [10]. In a first step, gp130-dependent genes were clustered based on their relative expression patterns across different time-points. Subsequently, a co-expression network was constructed for the group of highly correlated candidate genes (information on how co-expression networks are drawn: see Methods section).

**Figure 4:**
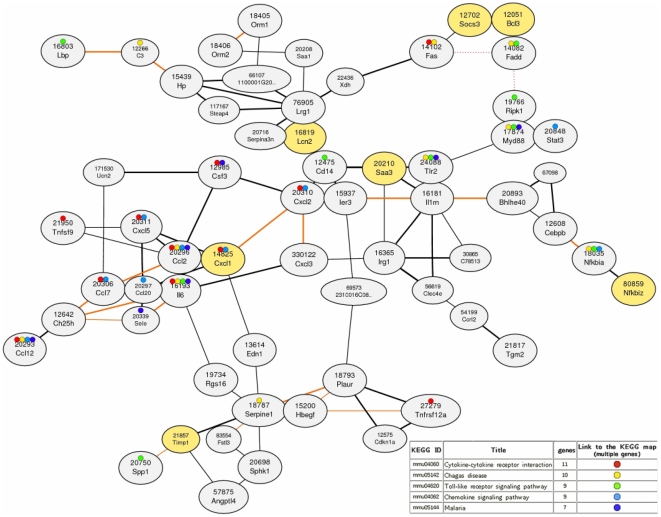
Co-expression network for highly correlated gp130 dependent genes. The co-expression network is drawn based on gene expression correlation patterns (Mutual rank scores) for the 8 candidate genes using the Coxpresdb tool [10]. A bold line indicates a high co-expression between the genes. Orange lines indicate genes co-expressed in mouse and men. Red dotted lines indicate protein-protein interactions.

The network analysis revealed that 7 of the 8 candidate genes (highlighted in yellow in Figure 4) are part of a distinct co-expression module. This co-expression module harbors genes involved in different functional aspects of atherogenesis.

Several members of the co-expression module are annotated with the KEGG classifications cytokine-cytokine interaction and Toll-like receptor signaling. This is in line with the results from the pathways analysis of overrepresented KEGG pathways for differentially expressed genes. Among these genes are various cytokines (*cxcl1*, *cxcl2*, *Il-6*, *ccl2*) and immune related genes (*cd14*, *myd88*, *nfkbiz*), which play an important role in the pathogenesis of atherosclerosis and as mediators of the inflammatory response. These genes include regulatory factors, such as *myd88*, *Il-6* and *cd14* which are prominent for mediating the innate immune response [11], [12].

## Discussion

Transcriptome profiling revealed 38 gp130 dependent regulated genes in response to IL-6 stimulation in hepatocytes. Within this group of genes group we found a cluster of 8 genes which showed a very specific correlated expression. Recently, an impaired acute phase response in IL-6 deficient mice following IL-6 injection in terms of the APPs haptoglobin, alpha 1-glycoprotein (orosomucoid (ORM2)) and SAA was observed [15]. Thus, the question arises, whether we could not detect haptoglobin or ORM2 to be differentially regulated in our study. Our time course was 0–12 hours, and these APPs were found to be differentially regulated after 24 hours of IL-6 injection. Moreover, in our whole genome microarray approach we also detected ORM2 and haptoglobin to be differential regulated, but both APPs did not reach a significance level of p<0.05. (ORM2 p = 0.06 after 3 hours and p = 0.09 after 6 hours of IL-6 stimulation, haptoglobin p = 0.05 after 12 hours of IL-6 stimulation). Additionally, Kopf et al used a less parallel technique which might be more sensitive than our microarray approach.

The combination of whole transcriptome profiling and co-expression network analysis revealed that the identified genes are not only highly correlated among themselves, but also involved in the same co-expression module as key mediators and markers of chronic inflammation. Besides well known markers of the acute phase response, such as *Il-6, cxcl1, stat3, saa3 or cd14*, genes involved in coagulation (*plaur*, *serpine1*) the complement system (*hp*, *c3*) and extracellular matrix formation (*timp-1*, *spp1*) are found in the same co-expression module with gp130-dependent genes.

These observations point to a link between different aspects of a joint early inflammatory response after IL-6 stimulation in hepatocytes (0 h–3 h). The small set of identified early response genes are likely to share a common role in the same physiological context. This is underlined by the pathway mapping, which revealed a functional link between these genes to activation of the Jak-STAT and cytokine-cytokine receptor KEGG pathways.

The IL-6/gp130 system has been shown to regulate a myriad of genes involved in the early activation of innate immune signaling [16]. A recent study established a link between the complement cascade and the gp130 system via cytokine interactions in macrophages [17]. Furthermore, Sander et. al showed that IL-6, which acts via gp130 is required for modulating the dynamics of the innate immune cell recruitment during acute inflammation [18]. The recruitment of innate immune cells plays a central role in chronic inflammation and in the formation of atherosclerotic plaques. A tight regulation of the innate immune cell activation is necessary to prevent an overflowing immune response which might lead to a chronic inflammatory state.

This interpretation is in line with the identification of a prominent inflammatory marker expressed in liver and adipose tissue. The adipokine LCN2 is central to cell defense, leads to sustained chronic inflammation and protects aging mice from insulin resistance [19],[20].

LCN2 was the only differentially regulated transcript measured in every time point which also showed significantly lower levels for soluble proteins. The functional role of this gene in the process of atherosclerosis remains enigmatic. However, several studies have shown a range of protective effects of LCN2. These include various environmental perturbations such as chronic inflammation, insulin resistance and thermal stresses [21], [22]. Of interest, the study of Hemdahl et al. [23] shows that LCN2 is increased in atherosclerosis and after myocardial infarction and colocalize with MMP-9 in atherosclerotic lesions with high-proteolytic activity, suggesting a modulating role for LCN2 in atherosclerosis. Further studies are needed to investigate the role of LCN2 in atherosclerosis in detail.

Taken together, these results suggest that multiple functional innate immune related factors can strongly affect gene expression in a gp130-dependent model of atherosclerosis. It seems likely that a combination of these co-regulated factors is involved in the complex interplay between the innate immune response and chronic inflammation observed in this model. The small cluster of 8 genes is associated with different regulatory parts of the early innate immune response. Dysregulation between these immune signaling pathways in the liver might have crucial effects on promoting atherosclerotic plaque formation via the recruitment of immune cells. These observations are in line with a recent study on hepatic acute-phase proteins in the gp130 model, promoting a close relationship between hepatocytes and the activation of innate immune cells [9].

In order to decipher this complex interplay between different cellular immune signaling pathways, we need to further elucidate phenotypic diversity caused by variations in the innate immune response. Targeting gp130-dependent immune cell recruitment might help to unravel the process of atherosclerotic plaque formation. Furthermore, the functional role of LCN2 might give important insights in the interplay between different chronic inflammatory phenotypes.

## Methods

### Mice

Mice lacking the gp130 gene in the liver on an atherosclerosis-prone background were generated as described previously [24]. In brief, albumin promoter–controlled expression of the Cre recombinase (*alb-cre^tg^*) ensured a hepatocyte-specific deletion of the floxed gp130 gene (*gp130flox/flox*). The resulting genotype, *alb-cre^tg^; gp130^flox/flo^* ^x^ is simplified by gp130–.Littermates with a floxed gp130 gene (*gp130^flox/flo^* ^x^) served as controls (simplified by gp130 flox).

To obtain mice susceptible for atherosclerosis, these mice were crossed with the atherosclerosis-prone apolipoprotein E (apoE)-mice (simplified by gp130–/apoE–). Animals were backcrossed on a C57BL/6 genetic background and housed in the animal facility at the Hannover Medical School during experiments. All of the experiments were approved by the governmental animal ethics committee and performed according to the guidelines of the Federation of European Animal Science Associations (03/683 and §4/39).

### Cell culture

Hepatocytes from gp130– and gp130flox mice were isolated as described previously [24]. Briefly, 10^6^ cells were cultured on collagen-coated 6 cm cell culture dishes (Nunc, Wiesbaden, Germany) in DMEM (Invitrogen, Carlsbad, CA) containing 4.5 g/L glucose supplemented with 10% FCS and penicillin/streptomycin for 24 hours. Cells were starved overnight and stimulated with 200 ng/mL IL-6 for 0–24 hours under serum-free conditions. Cell culture supernatants were collected and stored at −20°C.

### ELISA

Concentrations of IL-6 dependent released factors within the supernatant of hepatocytes were measured by ELISA (CXCL-1, LCN2 and TIMP-1, R&D-Systems, IL-24 and HAMP, Hölzel Diagnostik). In brief, supernatants of the hepatocytes were diluted (1∶10 for CXCL1-, LCS-2- and HAMP ELISA, 1∶5 for TIMP-1) or remain undiluted (IL-24), plasma samples from gp130–/apoE–mice and apoE–mice fed with a cholesterol rich diet were diluted (1∶100 for CXCL1-, LCN-2- and HAMP ELISA, 1∶10 for TIMP-1, and 1∶2 for IL-24 ELISA).

### Microarray data analysis and real-time PCR

Hepatocytes RNA samples from at least 5 experimental groups per time point were used for microarray analysis. RNA samples were prepared and hybridized on Illumina Mouse genome WG6V2 arrays. Gene expression levels were calculated using GenomeStudio version 1.6.0 software (Illumina). Array data were fit to a cubic spline normalization model in GenomeStudio, and the expressed gene targets were identified using a detection level ≥0.95. Differentially expressed genes were identified using the Illumina custom error model. Genes with a corrected p-value similar or <0.05 and/or fold change >1.5 or less than −1.5 were considered significantly regulated for each timepoint. Fold changes of selected genes were visualized in a heat map diagram. All data are MIAME compliant; Microarray data have been deposited in the Gene Expression Omnibus under accession no. GSE25278.

Gene expression was validated by real time PCR. Total RNA was isolated using TriFast-Reagent (peqLAB), and reverse transcribed (Superscript reverse transcriptase; Invitrogen) with oligo(dT) primers. Real time PCRs were performed in triplicates in a total volume of 25 µl using brilliant SYBR green PCR master mixture (Stratagene) on a 7300 Real-Time-PCR System (Applied Biosystems) in 96-well PCR plates (Applied Biosystems). An initial denaturation step at 95°C for 10 min was followed by 40 PCR cycles, each cycle consisting of 95°C for 15 sec, 60°C for 1 min, and 72°C for 1 min, and SYBR Green fluorescence emission was monitored after each cycle. For normalization, expression of GAPDH was determined in parallel as an endogenous control. Relative gene expression was calculated using the 2-delta delta CT method. Primer sequences for each gene are given in table 2.

**Table 2:** Primer sequences for real-time PCR.

Pimer	Sequence 5′ – 3′
SAA3 sense	ATG CTC GGG GGG AAC TAT GAT
SAA3 antisense	GAG TCC TCT GCT CCA TGT CC
HAMP sense	GAG CAG CAC CAC CTA TCT CC
HAMP antIsense	CAA TGT CTG CCC TGC TTT CT
SOCS-3 sense	GAG ATT TCG CTT CGG GAC TA
SOCS-3 antisense	AAC TTG CTG TGG GTG ACC AT
LCN2 sense	CCA GTT CGC CAT GGT ATT TT
LCN2 antisense	TCC TTC AGT TCA GGG GAC AG
TIMP1 sense	GAC CTA TAG TGC TGG CTG TGG
TIMP1 antisense	GTA GTC CTC AGA GCC CAC GA
CXCL1 sense	TGA AGC TCC CTT GGT TCA GA
CXCL1 antisense	AGG TGC CAT CAG AGC AGT CT
BCL3 sense	CAG TCG TCT CAG CTC CAA TG
BCL3 antisense	TGG GAA GGA AAA AGT TTT GG
NFΚBiz sense	CGC CCT CCA TGT TGC TGC CA
NFΚBiz antisense	TCT CCC ACT GGG CCA TCG GG

SAA3: Serum amyloid A3; HAMP: Hepcidin; SOCS3: supressor of cytokine signaling 3; CISH: cytokine inducible SH2 protein; LCN2: Lipocalin 2; TIMP1: Tissue inhibitor of metalloproteinase 1; CXCL1: Chemokine (C-X-C motif) ligand 1, BCL3: B-cell lymphoma 3-encoded protein, NFKBiz: nuclear factor of kappa light polypeptide gene enhancer in B-cells inhibitor, zeta.

### KEGG-Pathway Mapping

Genes differentially expressed, as derived from pair-wise gene-level comparison between time points, were selected for exploring which KEGG pathways were over-represented in this collection of genes. These included both up-and down-regulated genes. Hypergeometric testing was performed on this collection of genes to discover the over-represented KEGG pathways. This procedure used Fisher's exact test to find association between interesting genes (differentially expressed between time points) and membership to a KEGG pathway.

### Data on co-expression networks

Data on gene co-expression was obtained from the COXPRESdb, a expression database for mammals (www.coxpresdb.hgc.jp) [10]. The database contains a set of 2226 mice samples from 154 experiments. All samples were selected from Affymetrix Mouse Genome 430 2.0 Arrays and derived from NCBI's Gene Expression Omnibus (GEO) database (www.ncbi/nlm.nih.gov/geo). The robust-multi-array average (RMA) method was used for the normalization procedure of the raw expression data.

Pearson's correlation coefficients were computed to measure probe to probe expression patterns similarity for all possible gene pairs. The co-expression networks are drawn based on mutual rank information for the seven candidate genes. Dotted lines indicate protein-protein interactions derived from the HPRD database (www.hprd.org).

### Statistical analysis

Data are presented as mean±SEM of at least 5–8 independent experiments. For comparisons between three or more groups we used 2-way ANOVA followed by the Holm-Sidak method for multiple pair-wise comparisons. Differences were considered statistically significant at a value of P<0.05.

## Supporting Information

Table S1 **Transcriptome-wide analysis of 26,766 genes by microarray time-course analysis of isolated hepatocytes derived from gp130-mice and gp130flox used as contols.** Transcriptome profiling revealed a small number of 38 genes that are differentially expressed with a p-value of <0.05 and >1.5 fold after IL-6 stimulation in at least one of the five time points. N = 5 mice per genotype.
(DOC)
