# Supplementary material for: Gp130-Dependent Release of Acute Phase Proteins Is Linked to the Activation of Innate Immune Signaling Pathways
Source: PLoS One. 2011 May 4;6(5):e19427. doi: 10.1371/journal.pone.0019427 (PMC3087798; doi:10.1371/journal.pone.0019427)
Supplement: Table S1 — Transcriptome-wide analysis of 26,766 genes by microarray time-course analysis of isolated hepatocytes derived from gp130-mice and gp130flox used as contols. Transcriptome profiling revealed a small number of 38 genes that are differentially expressed with a p-value of <0.05 and >1.5 fold after IL-6 stimulation in at least one of the five time points. N = 5 mice per genotype. (DOC) [file pone.0019427.s001.doc]

Table S1. Transcriptome-wide analysis of 26,766 genes by microarray time-course analysis of isolated hepatocytes derived from gp130-mice and gp130flox used as contols

| **Target ID** | **0h p-value** | **Factor (fold change in gene expression between gp130- and gp130flox)** | **Regulation** |
| --- | --- | --- | --- |
| SAA3 | 0.00011419 | 15.5944227 | down |
| LCN2 | 5.27E-08 | 10.0300309 | down |
| TOR1AIP2 | 1.28E-05 | 4.33330387 | down |
| 9430065F12RIK | 0.00027413 | 3.447599 | up |
| 1700030C10RIK | 0.00462681 | 1.97057265 | down |
| 8030402P03RIK | 0.03058558 | 1.7804372 | down |
| MPG | 0.00410148 | 1.59958155 | down |
| **Target ID** | **1h p-value** | **Factor (fold change in gene expression between gp130- and gp130flox)** | **Regulation** |
| LCN2 | 0.000127198 | 10.8582266 | down |
| 8030402P03RIK | 0.000703735 | 2.42639883 | down |
| 9430065F12RIK | 0.001586687 | 3.488093 | up |
| TOR1AIP2 | 0.00469822 | 3.88161238 | down |
| SAA3 | 0.005610913 | 16.4139439 | down |
| SOCS3 | 0.008155542 | 9.62166645 | down |
| 1700030C10RIK | 0.01720031 | 2.03685319 | down |
| IL24 | 0.01789633 | 2.10886199 | down |
| MPG | 0.02798741 | 1.58800207 | down |
| HAMP | 0.04263411 | 3.76679898 | down |
| **Target ID** | **3h p-value** | **Factor (fold change in gene expression between gp130- and gp130flox)** | **Regulation** |
| LCN2 | 0.000199196 | 13.4574505 | down |
| SOCS3 | 0.000199196 | 9.18362193 | down |
| FGA | 0.000990348 | 8.11673159 | down |
| TGM1 | 0.003445756 | 4.45473191 | down |
| IL13RA1 | 0.01385522 | 3.68176041 | down |
| IL24 | 0.01602176 | 2.22628841 | down |
| LOC666238 | 0.02436866 | 3.50126448 | down |
| LOC666185 | 0.02436866 | 3.18015482 | down |
| 9430065F12RIK | 0.02436866 | 3.556646 | up |
| MPG | 0.02601274 | 1.71690178 | down |
| CISH | 0.03320469 | 3.45863627 | down |
| LOC637082 | 0.03719956 | 1.66117648 | down |
| BCL3 | 0.03797411 | 3.72123209 | down |
| TIMP1 | 0.03936021 | 4.12545169 | down |
| ARNTL2 | 0.04813703 | 1.53386184 | down |
| TOR1AIP2 | 0.04977062 | 3.79687623 | down |
|  |  |  |  |
| **Target ID** | **6h p-value** | **Factor (fold change in gene expression between gp130- and gp130flox)** | **Regulation** |
| LCN2 | 5.88E-06 | 12.9505261 | down |
| SOCS3 | 9.24E-05 | 11.048437 | down |
| FGA | 0.000146328 | 7.42478691 | down |
| SAA3 | 0.000294599 | 30.2224554 | down |
| BCL3 | 0.000602509 | 3.59756747 | down |
| 9430065F12RIK | 0.000971359 | 3.417743 | up |
| IL24 | 0.000993929 | 2.30844681 | down |
| IL13RA1 | 0.00169273 | 3.38599343 | down |
| SH3PXD2B | 0.00169273 | 3.26932005 | down |
| 8030402P03RIK | 0.00169273 | 1.95376646 | down |
| SERPINA7 | 0.00344327 | 5.51113884 | down |
| 1700030C10RIK | 0.003641485 | 2.12758262 | down |
| TOR1AIP2 | 0.003689556 | 3.89240005 | down |
| HAMP | 0.008127714 | 5.11570706 | down |
| RDH10 | 0.008744544 | 2.125722 | down |
| MPG | 0.01185227 | 1.74518242 | down |
| ID1 | 0.01467392 | 3.04779925 | down |
| TIMP1 | 0.01835142 | 3.86268913 | down |
| NFKBIZ | 0.01841593 | 2.90465724 | down |
| LOC666185 | 0.02368907 | 3.20227182 | down |
| RASL12 | 0.02605664 | 1.68195281 | down |
| LOC666238 | 0.02708476 | 3.06779427 | down |
| ARNTL2 | 0.02708476 | 1.51081821 | down |
| CISH | 0.02828519 | 2.82995507 | down |
| OSMR | 0.0301275 | 2.47420517 | down |
| FOS | 0.04220745 | 2.33680349 | down |
| SPSB1 | 0.0454298 | 2.26334724 | down |
| C920030L09RIK | 0.04598212 | 2.95605062 | down |
|  |  |  |  |
|  |  |  |  |
|  |  |  |  |
|  |  |  |  |
| **Target ID** | **12h p-value** | **Factor (fold change in gene expression between gp130- and gp130flox)** | **Regulation** |
| LCN2 | 1.46E-06 | 14.5701231 | down |
| FGA | 0.000191225 | 7.48086955 | down |
| SAA3 | 0.001422926 | 33.2817245 | down |
| 1700030C10RIK | 0.001422926 | 2.18887112 | down |
| 8030402P03RIK | 0.001422926 | 1.95263798 | down |
| BCL3 | 0.001428132 | 3.08831468 | down |
| 9430065F12RIK | 0.002247434 | 3.456372 | up |
| HAMP | 0.002825127 | 5.6941901 | down |
| TOR1AIP2 | 0.003549828 | 4.22683567 | down |
| LOC666185 | 0.003565194 | 3.12499316 | down |
| PRELP | 0.003565194 | 2.82279349 | down |
| NFKBIZ | 0.003810619 | 3.24283763 | down |
| SOCS3 | 0.004635807 | 7.79513755 | down |
| SH3PXD2B | 0.005528733 | 3.25965641 | down |
| ID1 | 0.007464438 | 3.06659385 | down |
| LOC666238 | 0.008551609 | 2.96645943 | down |
| SERPINA7 | 0.01286673 | 5.8481721 | down |
| MPG | 0.01488995 | 1.63748907 | down |
| IL13RA1 | 0.01641721 | 2.87587549 | down |
| C920030L09RIK | 0.02402725 | 2.94789768 | down |
| VNN3 | 0.02536883 | 2.64020199 | down |
| PDZRN3 | 0.02771287 | 3.58940508 | down |
| FOS | 0.03094408 | 2.26031353 | down |
| RDH10 | 0.03094408 | 2.15878838 | down |
| BMPER | 0.03094408 | 1.95356417 | down |
| IL24 | 0.03094408 | 1.8935571 | down |
| 4833442J19RIK | 0.03111047 | 2.81292844 | down |
| OSMR | 0.03181613 | 2.186286 | down |
| IGF2BP2 | 0.03196449 | 2.63693053 | down |
| 1700112C13RIK | 0.0320155 | 1.56077266 | down |
| CXCL1 | 0.04660054 | 2.93644965 | down |
